# Variation of counting efficiency in determination of ^131^I activity in the thyroid gland as a result of its position relative to the detector

**DOI:** 10.1007/s10967-014-3051-z

**Published:** 2014-03-09

**Authors:** R. Kierepko, P. Janowski, M. Grochowska

**Affiliations:** 1 Institute of Nuclear Physics Polish Academy of Sciences, Ul. W. E. Radzikowskiego 152, 31-342 Kraków, Poland; 2 AGH University of Science and Technology, Faculty of Physics and Applied Computer Science, Al. A. Mickiewicza 30, 30-059 Kraków, Poland

**Keywords:** Thyroid phantom, Whole body spectrometry, KI, ^131^I, Activity concentration

## Abstract

The appropriate determination of the ^131^I which was absorbed into the human body, especially by thyroid, depends not only on individual features of each measurement subjects but also on reproducibility of their position or the thyroid’s position in the human neck. Possible uncertainties caused by changes of the thyroid position relative to detector were studied in a series of measurements. The research has shown that the dispersion of the results can reach up even to the level of 50 %.

## Introduction

Determination of the body burden with gamma emitters is one of the most important measurements in radiation protection for e.g. medical staff who work in domain of nuclear medicine, research staff or student who work with radioisotopes, etc. The whole body spectrometry is a well known technique that enables to get information about activity concentration and distribution of radionuclides in measurement subject. The most important aspect of activity determination when measuring human body is not to underestimate uncertainties connected with individual features (e.g. size, shape, etc.) and the position of each organ in human body [1–3]. The thyroid was chosen as an object of measurements. A number of them, simulating different positions of the thyroid relative to the detector was done. It took place in the Institute of Nuclear Physics Polish Academy of Sciences (IFJ PAN) The thyroid is one of the larger and an odd endocrine glands, weighing 2–3 grams in neonates and 18–60 grams in adults, and is increased in pregnancy [4]. It is composed of two lobes connected by the isthmus of thyroid and, in some people, a small pyramidal lobe [5]. Thyroid volume measured by the ultrasound probe is: in women − to 18 cm^3^, and in men − to 25 cm^3^ [4].

## Method

The series of the measurements was performed at IFJ PAN using the Whole Body Spectrometer (WBS) with 2 HPGe detectors (X, Y 30 % relative efficiency each). One of them is pointing at patient’s thyroid (X) and the other is located over abdomen (Y). The distance between centers of their windows is 52 cm. The main part is shielding, made of 18 t of XIX century steel, free from Co-60 contamination [6]. The outer dimensions of the chamber are 2.0 m × 1.5 m × 1.5 m with the width of the walls 15 cm.

**Fig. 1:**
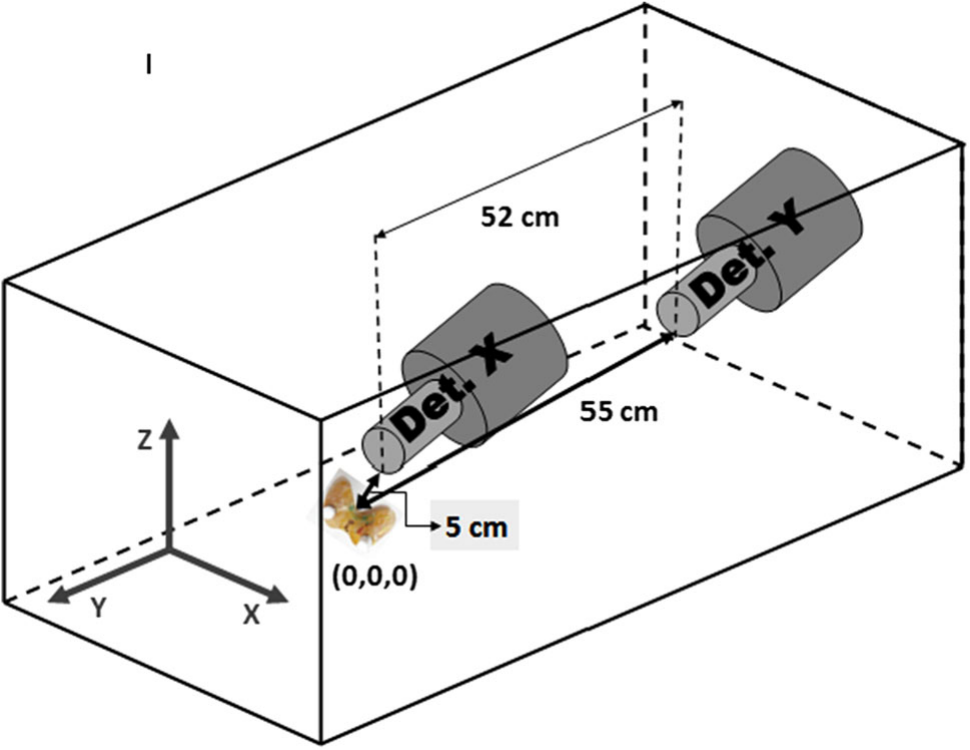
A scheme of the Whole Body Spectrometer (WBS) chamber with the (0,0,0) position of the thyroid phantom and distances between this point and detectors of the WBS

The conditions of real measurements were recreated. During one hour long measurement the patient is lying on a slightly curved bed so that the body is forming a light arc around detectors. A special phantom human thyroid and holder for it were designed. The anatomical shape of the phantom was made of an epoxy resin and consisted of two glands with small air cavities inside (16 cm^3^). Construction of the holder makes it possible to move the phantom in three directions (x,y,z) (Fig. 1.). The ranges of shifts along x, y, z axis were respectively (−5,5), (−2,2), (−2,2) (in cm) with a step of 1 cm. At the beginning of the measurement the point (0,0,0) was chosen. It was about 5 cm from X and 55 cm from Y detector (Fig. 1.). The mean time of each step was about 10 min with the dead time not higher than 0.2 % for both detectors.

The measurements were performed for thyroid phantom filled by standard solution of ^131^I with chemical composition 50 mg of KI in 1 dm^3^ of solution and activity amounted to 6 kBq on first day of the procedure. The half life time correction was done for all results.

## Results

**Fig. 2:**
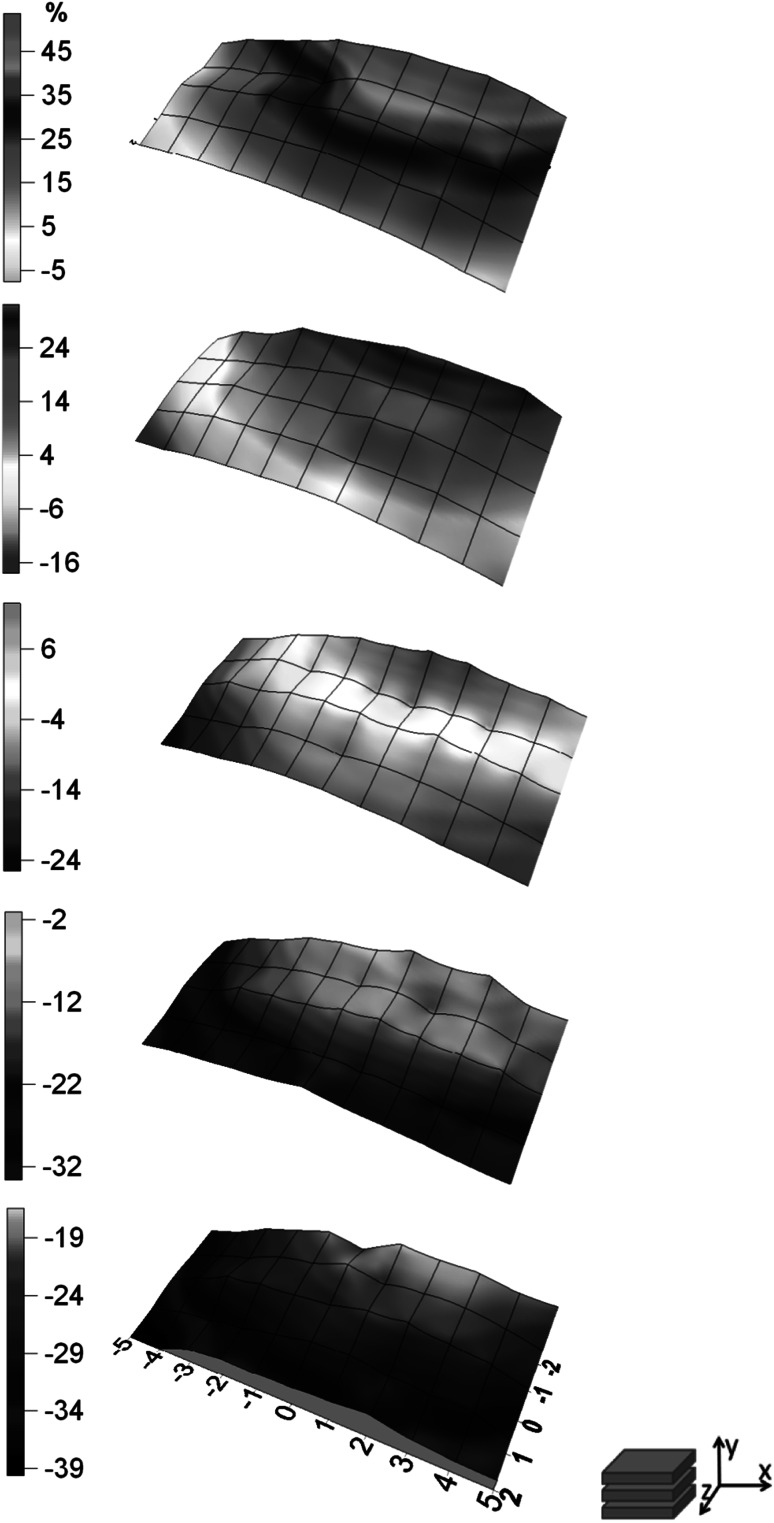
The distribution of relative differences of efficiency versus thyroid phantom position shown in (x,y) plane for X detector

**Fig. 3:**
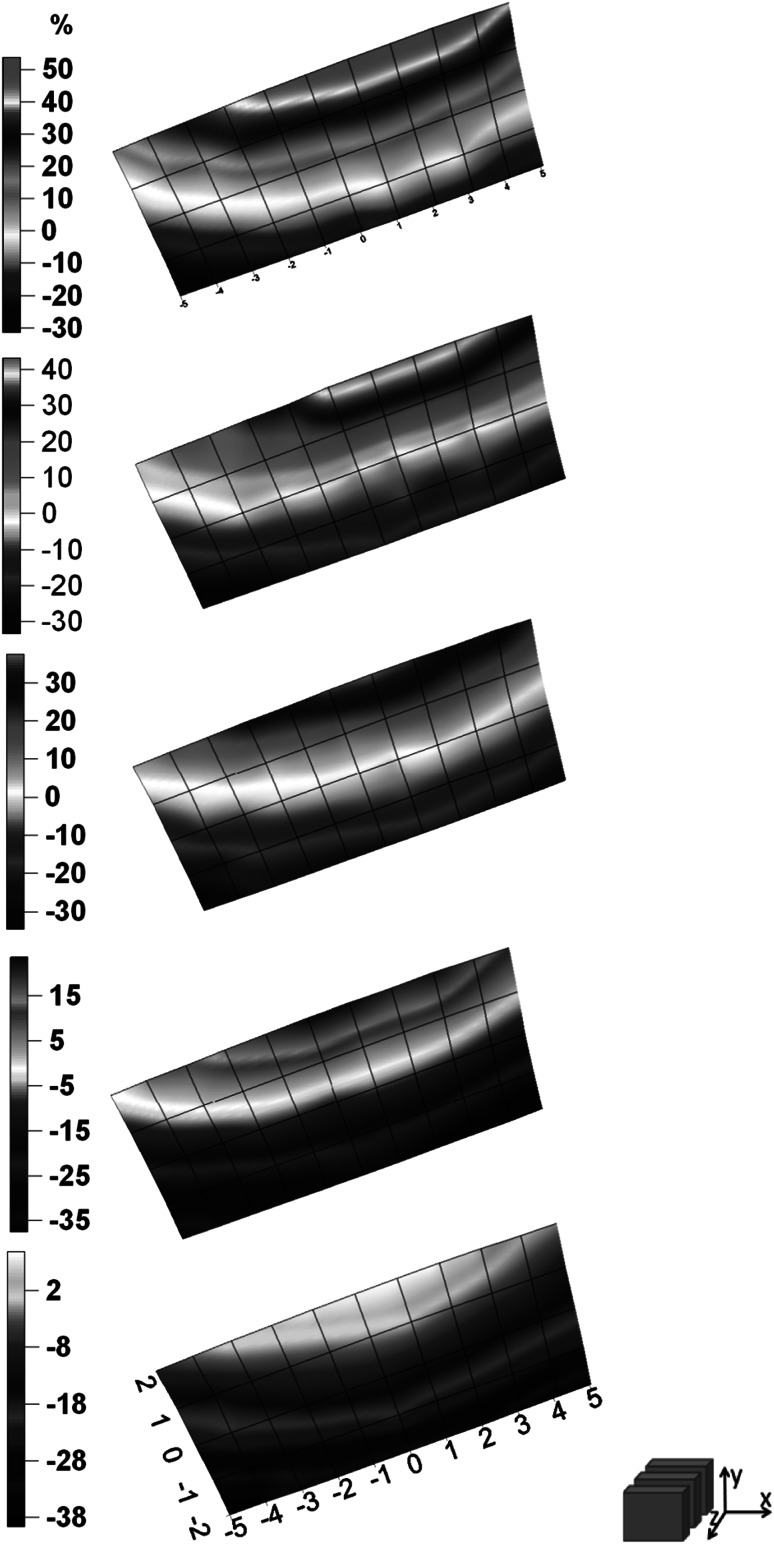
The distribution of relative differences of efficiency versus thyroid phantom position shown in (x,z) plane for X detector

**Fig. 4:**
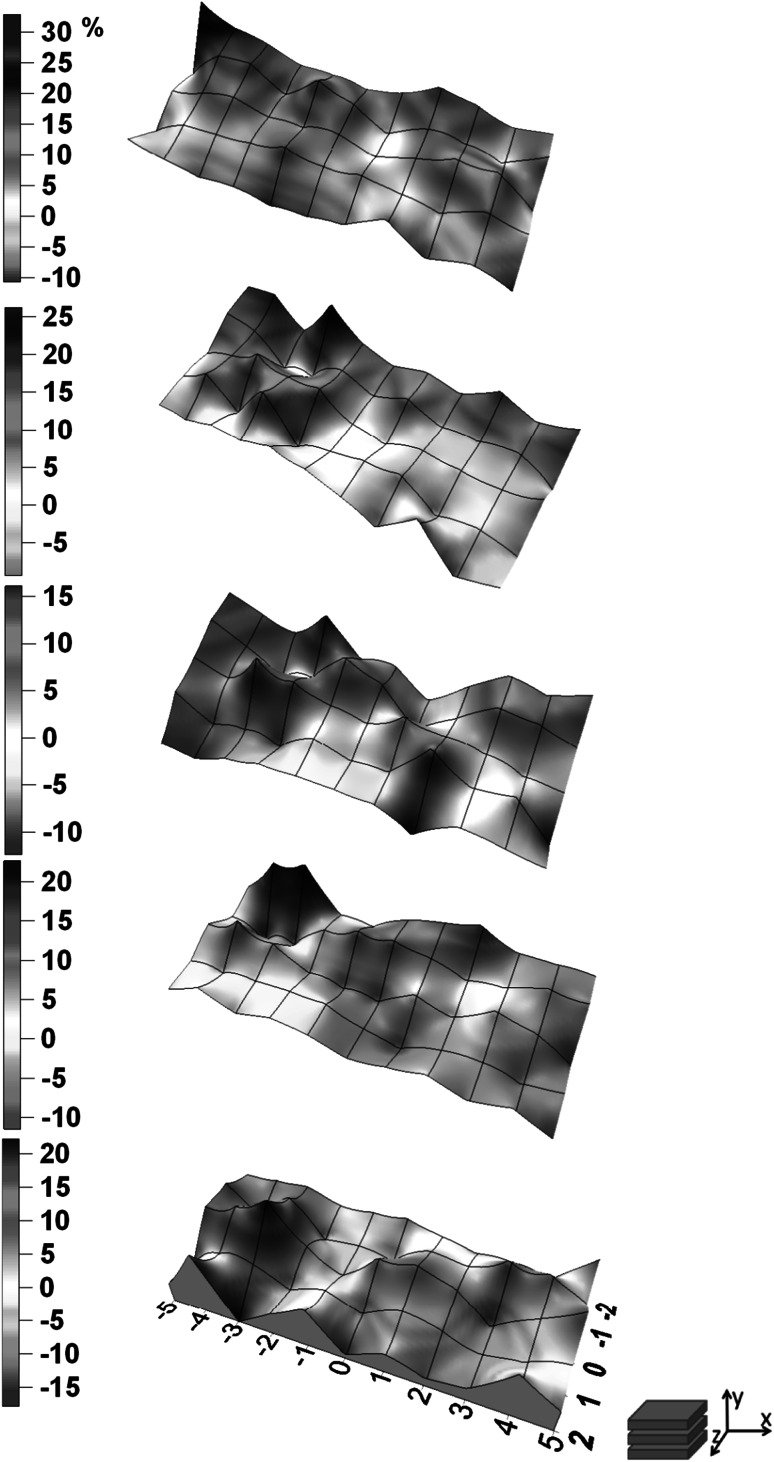
The distribution of relative differences of efficiency versus thyroid phantom position shown in (x,y) plane for Y detector

**Fig. 5:**
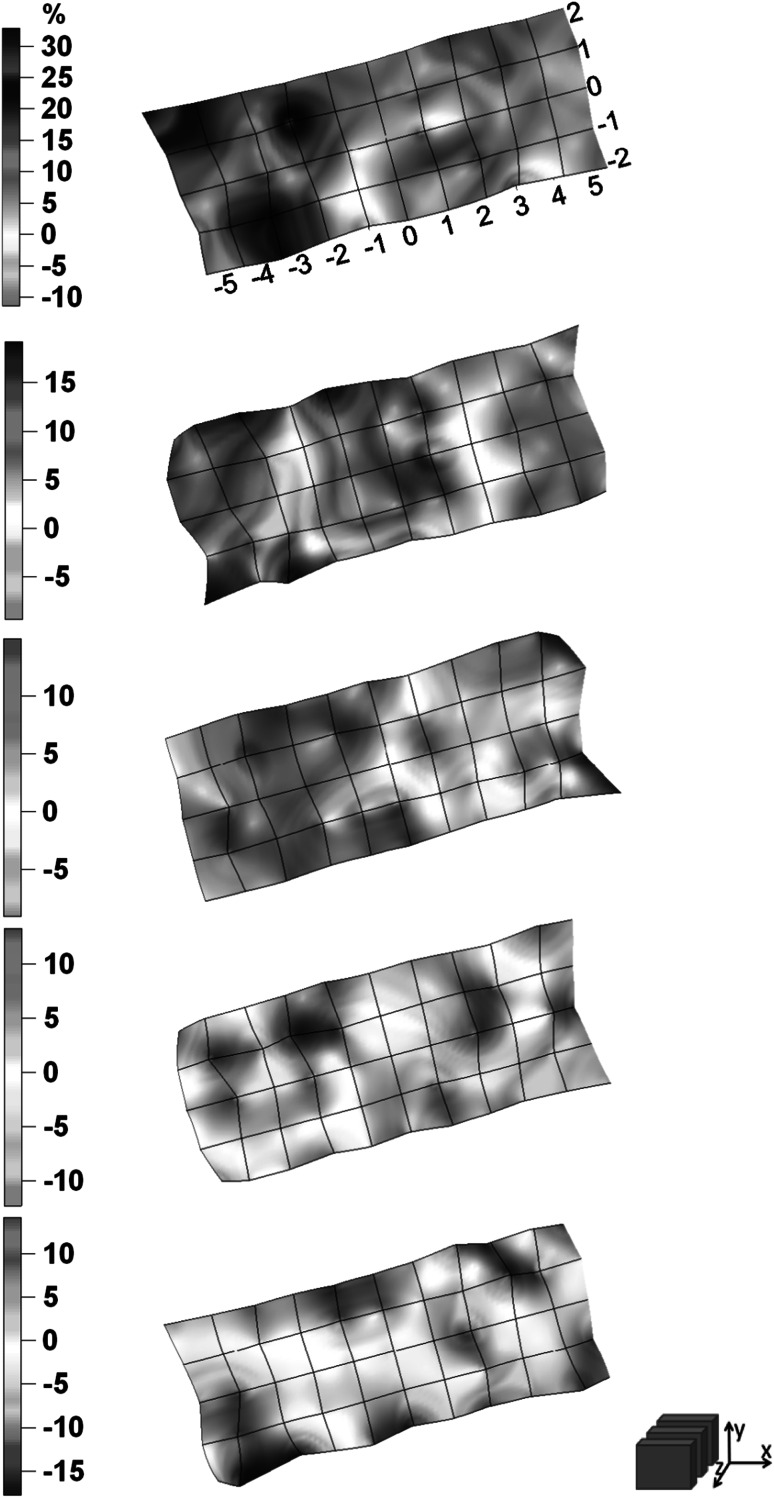
The distribution of relative differences of efficiency versus thyroid phantom position shown in (x,z) plane for Y detector

The total number of measurements which were done during the experiment equals 275. Uncertainties which resulting from counting statistics for energy peak 364.5 keV were not higher than 2 and 10 % for X and Y detector respectively. Figs. 2, 3, 4, 5. show the distribution of relative difference of efficiency (Eq.1) connected to shift of the thyroid phantom. Results are presented as surfaces bordered by prescribed conditions for experiment

1 $$ \delta = \frac{{\varepsilon_{{\left( {0,0,0} \right)}} - \varepsilon_{{\left( {x_{i} ,y_{i} ,z_{i} } \right)}} }}{{\varepsilon_{{\left( {0,0,0} \right)}} }} \cdot 100\% $$

where:

$\delta$–relative difference of efficiency,

$\varepsilon_{{\left( {0,0,0} \right)}}$–efficiency of measurement for thyroid phantom geometry at (0,0,0) point,

$\varepsilon_{{\left( {x_{i} ,y_{i} ,z_{i} } \right)}}$–efficiency of measurement for thyroid phantom geometry at (x_i_, y_i_, z_i_) point, where: *i* = 1, 2, 3…274.

The displacement of the phantom along x, y, z axis resulted in considerable changes of efficiency which reached up to the level of about 50 % for X and 30 % for Y detector. The lower than anticipated value for Y detector is due to the fact that the Y detector is located further and it is less sensitive to the influences of the small displacement of the thyroid.

The research allowed to determine the area in WBS at IFJ PAN which is predestined for thyroid measurements characterized by relatively small uncertainties dependent of distance changes.

The two detectors system which was calibrated for thyroid gland geometry has the advantage of giving more information about distribution of ^131^I in human body. The higher results for Y detector do not have any physical meanings besides the sole information, that ^131^I is observed somewhere else than in thyroid.

## Conclusion

The results of the presented studies confirmed that the uncertainties dependent of the position of the thyroid during the measurements are not negligible. The described series of the measurements allowed us to find the optimal area for the thyroid position. In this space small shifts of this organ result in the smallest observed changes of relative differences of efficiency.
